# Ocular direct current stimulation affects retinal ganglion cells

**DOI:** 10.1038/s41598-021-96401-9

**Published:** 2021-09-02

**Authors:** Maren-Christina Blum, Alexander Hunold, Benjamin Solf, Sascha Klee

**Affiliations:** 1 Institute for Biomedical Engineering and Informatics, Technische Universität Ilmenau, Ilmenau, Germany; 2 Department of General Health Studies, Division Biostatistics and Data Science, Karl Landsteiner University of Health Sciences, Krems, Austria

**Keywords:** Electrophysiology, Retina, Biomedical engineering

## Abstract

Ocular current stimulation (oCS) with weak current intensities (a few mA) has shown positive effects on retinal nerve cells, which indicates that neurodegenerative ocular diseases could be treated with current stimulation of the eye. During oCS, a significant polarity-independent reduction in the characteristic P50 amplitude of a pattern-reversal electroretinogram was found, while no current stimulation effect was found for a full field electroretinogram (ffERG). The ffERG data indicated a trend for a polarity-dependent influence during oCS on the photopic negative response (PhNR) wave, which represents the sum activity of the retinal ganglion cells. Therefore, an ffERG with adjusted parameters for the standardized measurement of the PhNR wave was combined with simultaneous oCS to study the potential effects of direct oCS on cumulative ganglion cell activity. Compared with that measured before oCS, the PhNR amplitude in the cathodal group increased significantly during current stimulation, while in the anodal and sham groups, no effect was visible (α = 0.05, p_cathodal_ = 0.006*). Furthermore, repeated-measures ANOVA revealed a significant difference in PhNR amplitude between the anodal and cathodal groups as well as between the cathodal and sham groups (p* ≤ 0.0167, p_cathodal − anodal_ = 0.002*, p_cathodal − sham_ = 0.011*).

## Introduction

Transcranial direct current stimulation (tDCS) is an established method for research on the human nervous system and for the treatment of neuronal disorders^1,2^. In tDCS, low currents (e.g., magnitude of a few mA) are applied for several minutes via electrodes attached to the participant's head. The influence of tDCS on neuronal activity is demonstrated by polarity-dependent effects on visual evoked potentials (VEPs)^3–7^. Antal et al.^3^ showed a significant reduction in the N75 amplitude after cathodal stimulation (according to the international 10–20 system for EEG electrode positions, Oz-Cz), while anodal stimulation slightly increased the amplitude (not statistically significant). Ding et al.^5^ found a similar result for the P100 amplitude, but in their study, anodal stimulation produced a significant increase, while cathodal stimulation only showed a trend for amplitude reduction. The inverse result was found by Accornero et al.^4^, where cathodal stimulation increased and anodal stimulation decreased the P100 amplitude. However, in there study, different electrode positions (Oz to neck) were used relative to previous studies^4^.

Some existing research has focused on the application of current stimulation methods to the retinal system for the treatment of neurodegenerative retinal diseases. Chow et al.^8^ found visual improvements in patients with an implanted retinal prosthesis even in areas far from the prosthesis. These improvements were attributed to the weak current pulses applied by the prosthesis to the underlying retinal cells. Subsequently, the effects of ocular current stimulation (oCS) on retinal cells were investigated in animal studies. In these studies, oCS showed an effect on the survival rate of retinal ganglion cells and photoreceptors, and in general, positive effects on the development, functionality, and stability of retinal nerve cells were found^9–14^. Human studies have mainly investigated the effect of oCS on neurodegenerative eye diseases such as glaucoma^15,16^, retinitis pigmentosa^17–20^, Stargardt disease^21^, macular degeneration^22–24^, retinal artery occlusions^25,26^, or optic neuropathy^27–29^. However, the treatment outcome from these studies is partly contradictory, and reproducible treatment success has not been achieved thus far.

This is partly hampered by the limited knowledge about which retinal cells are specifically influenced by oCS. Here, we aim to address this topic by analyzing the effects of oCS on the electroretinogram (ERG). To advance the understanding of electrical stimulation effects on the visual system, Blum et al.^30^ were interested whether an oCS shows similar effects in the ERG as tDCS does on the VEP. Blum et al.^30^ found a significant reduction in the P50 amplitude of a pattern-reversal electroretinogram (PERG) during anodal and cathodal oCS, while no effect was found for sham stimulation. This polarity-independent oCS effect on the P50 amplitude could originate from modulations of preganglion cell activity or by changes in the local ON and OFF responses of ganglion cells. A follow-up study^31^ was conducted to further investigate the oCS effect on full field ERG (ffERG), with which preganglion cells (especially photoreceptors and bipolar cells) can be studied^32^. In this study, no oCS effect was found on ffERG amplitudes or latencies. Therefore, it was concluded that the changes in the P50 amplitude in the PERG study did not result from the modulation of preganglion cells. However, the ffERG responses indicated a negative wave after the characteristic b-wave, which showed an increasing amplitude during cathodal oCS and no effect for anodal and sham stimulation^33^. This negative wave corresponds to the photopic negative response (PhNR), which is generated in retinal ganglion cells and reflects their sum activity^34–38^. The PhNR is most distinctive for a light-adapted ffERG in response to a brief red flash (≤ 5 ms) on a blue background^37^. Investigation of the oCS effect on the PhNR wave, recorded according to the standard of the International Society of Clinical Electrophysiology of Vision (ISCEV)^35^, can provide insights into the potentially polarity-dependent affectability of cumulative retinal ganglion cell activity. The results can be related to the mentioned VEP studies, since the VEP reflects an electrophysiological sum response and shows polarity-dependent effects on tDCS.

To advance the understanding of oCS effects on retinal cell types, this study focuses on the PhNR wave. First, a new measurement and stimulation setup was developed to enable simultaneous oCS and PhNR measurement. Subsequently, we conducted a study in which the PhNR was measured before (ERG 1) and during (ERG 2) oCS for three different current applications (i.e., cathodal polarity, anodal polarity, or sham stimulation). Given the current stimulation effects reported in VEP and previous ERG studies, we hypothesize that the characteristic PhNR wave will be affected during oCS in a polarity-dependent manner. The study results can confirm the effects of oCS on retinal ganglion cells. Providing insights into which and how retinal cells respond to oCS can potentially guide therapy of neurodegenerative retinal diseases with oCS and might generalize to other neuronal tissues.

## Results

Current stimulation, visual stimulation, and ERG measurements were performed and evaluated for all 17 volunteers (mean age: 25.2 ± 3.1 years, 9 females) with the newly developed measurement and stimulation setup. The requirements were therefore met by the following setup.

An electrophysiological full field stimulator (RETI-port/scan 21 Q450 stimulator, Roland Consult Stasche & Finger GmbH, Brandenburg a.d. Havel, Germany) with freely adjustable stimulation parameters was used for visual stimulation. The visual full field stimulus for measuring the PhNR wave was designed according to the ISCEV^35^ (red flash: 625 nm, luminance: 1.505 cds/m^2^, duration: ≤ 5 ms, frequency: 2 Hz, blue background illumination: 455 nm, luminance background: 10 cd/m^2^). The volunteers placed their heads in a height-adjustable chin rest to reduce movement artifacts. Ring-shaped sintered Ag/AgCl recording electrodes (EASYCAP GmbH, Herrsching, Germany) were attached to the skin of the lower eyelid (active), ipsilateral earlobe (reference), and forehead (ground). A galvanically isolated amplifier system (Cubias-M, neuroCare Group GmbH, Munich, Germany) with a 24-bit analog-to-digital converter, an input impedance of  ≥ 10 GΩ, an internal noise level of  ≤ 0.9 µV, and a sampling rate of 2000 samples-per-second recorded the ERG. The amplifier system allowed operation with different dynamic ranges and gain settings and thus enabled sensitivity optimization to different measurement conditions. In this study, we choose a dynamic range of  ± 170 mV. The applied current stimulator (DC-Stimulator MC, neuroCare Group GmbH, Munich, Germany) was battery powered, and the impedance between the stimulation electrodes was monitored by recording the applied current and adjusted voltage continuously during the entire oCS. A ring rubber electrode (outer/inner diameter: 75 mm/30 mm; thickness: 2 mm) spread with Ten20 conductive gel (Weaver and Company, Aurora, Colorado, United States) surrounded the eye and applied the current. The electrode had a cutout in the area of the lower eyelid to allow the placement of the ERG recording electrode without connection. The counter electrode was a rubber patch (25 cm^2^, thickness: 2 mm) inserted into a saline-soaked (10 ml) sponge and positioned at the ipsilateral tempus. For compatibility with the previous studies the current strength was set to 800 µA with a duration of 5 min. Linear current slopes over 5 s at the beginning and the end of the current stimulation were meant to avoid transient current sensations or skin irritation under the stimulation electrodes.

Table 1 summarizes the mean amplitudes and latencies of the measured PhNR waves. The graphical and data distribution analyses of the latencies showed no indications of common current effects, whereupon a statistical evaluation of the latencies was waived.

**Table 1:** Measured mean values with standard deviation for the different current applications (e.g., cathodal polarity, anodal polarity, and sham stimulation) and ERG measurements (ERG 1 before, ERG 2 during ocular current stimulation) for the PhNR amplitude and latency.

Current application	Measurement	PhNR	
		Amplitude in µV	Latency in ms
Cathodal	ERG 1	-11.29 ± 2.88	68.71 ± 5.21
	ERG 2	-12.52 ± 3.07	69.00 ± 5.42
Anodal	ERG 1	-11.86 ± 3.31	68.03 ± 4.12
	ERG 2	-11.08 ± 3.02	69.12 ± 4.93
Sham	ERG 1	-12.08 ± 3.67	69.09 ± 5.21
	ERG 2	-11.71 ± 3.43	68.62 ± 4.80

**Figure 1:**
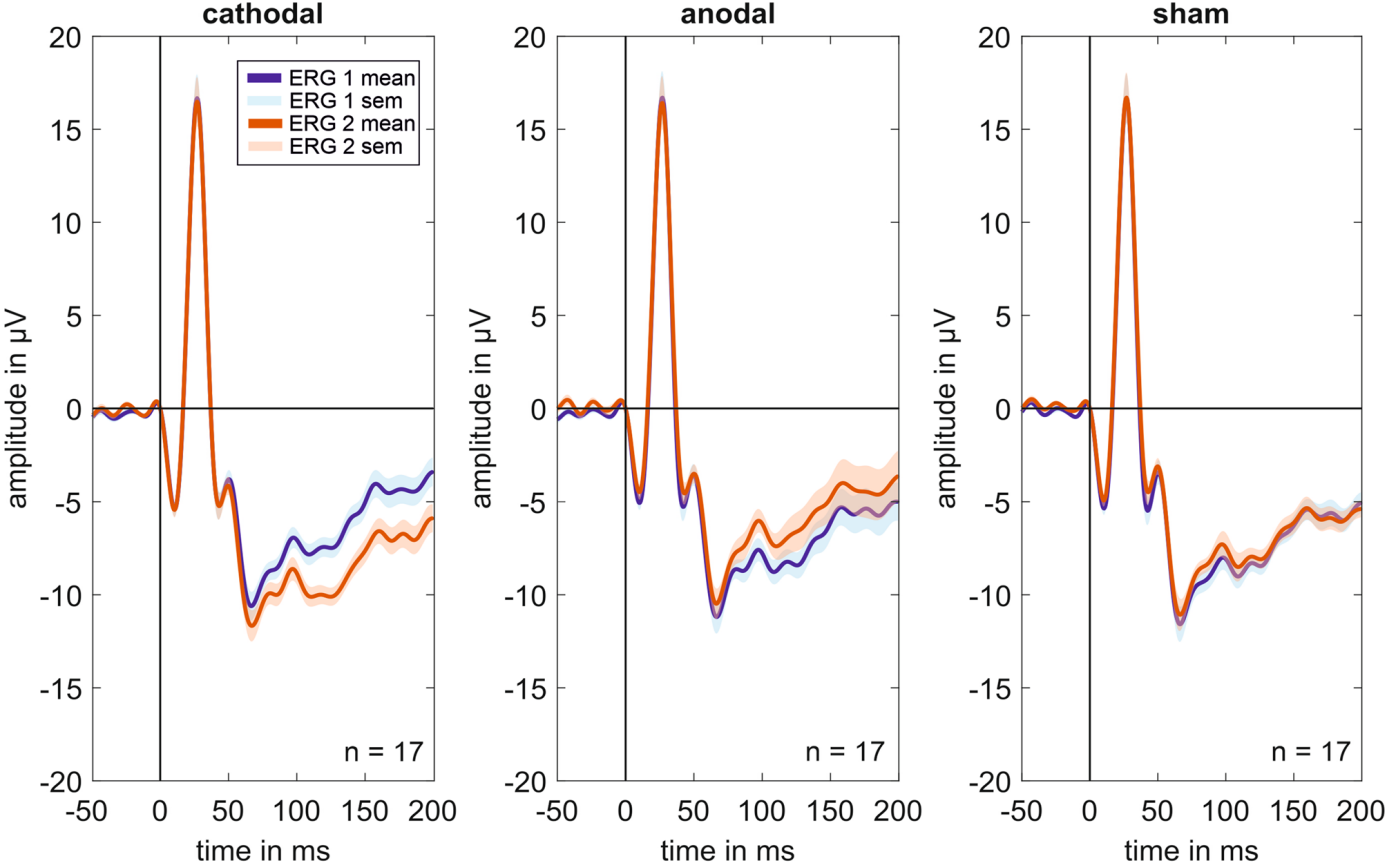
Grand mean signals. Grand mean curves for every stimulation group (i.e., cathodal polarity, anodal polarity, and sham stimulation; n = 17 for each curve) for the different ERG measurements (ERG 1 and ERG 2). ERG 1 (blue curve) was done before and ERG 2 (orange curve) during the current stimulation. An increasing current effect is visible for the photopic negative response (PhNR) amplitude in the cathodal stimulation group. In the anodal and sham stimulation group, a decreasing PhNR amplitude is seen for the ERG 2 in comparison to the ERG 1 measurement. The effect is more pronounced in the anodal group. *sem* standard error of the mean.

Figure 1 shows the grand mean signals across all volunteers for the different stimulation groups and ERG measurements. An increasing PhNR amplitude in the cathodal stimulation group was visible, while the anodal and sham stimulation groups showed only a small decreasing effect during oCS.

**Figure 2:**
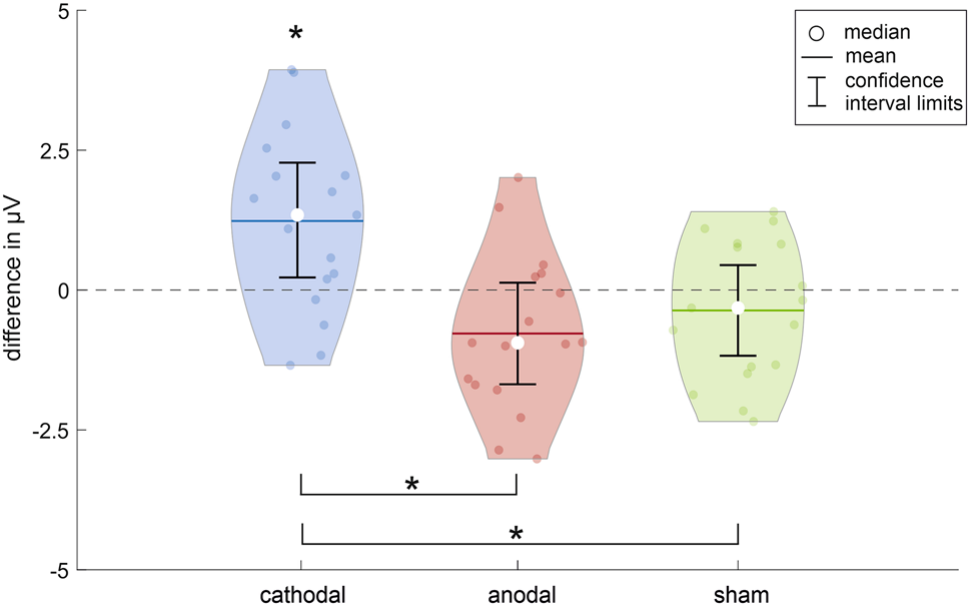
Data distribution and statistical test results. The violin plots and confidence intervals show the data distribution of the photopic negative response (PhNR) amplitude differences between the ERG 1 and ERG 2 measurement (ERG 1 before, ERG 2 during ocular current stimulation) for the three current stimulation groups (blue: cathodal polarity, red: anodal polarity, green: sham stimulation). The t-test for related samples revealed a significant difference within the cathodal current stimulation group, as indicated by not containing zero in the associated confidence interval (α = 0.05, Bonferroni corrected p* ≤ 0.0167). Furthermore, ANOVA for repeated measures found a significant difference between the amplitude changes from ERG 1 to ERG 2 for the cathodal to the anodal as well as for the cathodal to the sham stimulation group (ANOVA: α = 0.05, p = 0.001*; post hoc t-test for related samples Bonferroni corrected p* ≤ 0.0167). Significant results are marked with *.

Figure 2 shows the data distribution including the confidence intervals for the PhNR amplitude difference between the ERG 1 and ERG 2 measurements for all current application groups. The data distribution of the cathodal stimulation group showed an increasing PhNR amplitude during the oCS. The mean amplitude changed by 1.23 ± 1.60 µV (10.9%). The confidence interval [0.20 µV; 2.27 µV] excluded zero, which represents a significant change between the two measurements. Furthermore, the t-test confirmed the significant difference between the ERG 1 and ERG 2 measurements (p_cathodal_ = 0.006*, p* ≤ 0.0167). The effect size according to Cohen’s d^39^ was d_cathodal_ = 0.77. For the anodal and sham stimulation groups, the data distribution showed a small decreasing effect, although this was more pronounced in the anodal group. The mean PhNR amplitude of the anodal stimulation group changed from the ERG 1 to the ERG 2 measurement by -0.78 ± 1.40 µV (-6.6%), and that of the sham stimulation group changed by -0.37 ± 1.25 µV (-3.0%). The confidence interval of both groups included zero, indicating no significant change in the PhNR amplitude during oCS (confidence interval: anodal [-1.69 µV; 0.13 µV], sham [-1.18 µV; 0.45 µV]). These results were confirmed by the t-test (p_anodal_ = 0.036, p_sham_ = 0.246, p* = 0.0167). The effect size of the anodal stimulation was d_anodal_ = 0.55 and that of the sham stimulation was d_sham_ = 0.29. Table 2 summarizes the statistical test results.

**Table 2:** Results of statistic evaluation (t-test for related samples, effect size after Cohen’s d) whether there is a difference between ERG 1 and ERG 2 measurement (ERG 1 before, ERG 2 during ocular current stimulation) within a current application group.

	t-test p-value	confidence interval		effect size
		lower limit	upper limit	
Cathodal	0.006*	0.20 µV*	2.27 µV*	0.77
Anodal	0.036	-1.69 µV	0.13 µV	0.55
Sham	0.246	-1.18 µV	0.45 µV	0.29

Significant results are marked with *.

The second aim of the study was to analyze the difference between the three current application groups. Repeated measures ANOVA of the differences in the PhNR amplitude between the ERG 1 and ERG 2 measurements for all three current application groups revealed a significant difference between the groups in relation to the applied current stimulation (p = 0.001*, p* ≤ 0.05). The post hoc t-test for related samples found a significant difference between the cathodal and anodal as well as between the cathodal and sham stimulation groups (p_cathodal − anodal_ = 0.002*, p_cathodal − sham_ = 0.011*, p_anodal − sham_ = 0.305, p* ≤ 0.0167).

**Figure 3:**
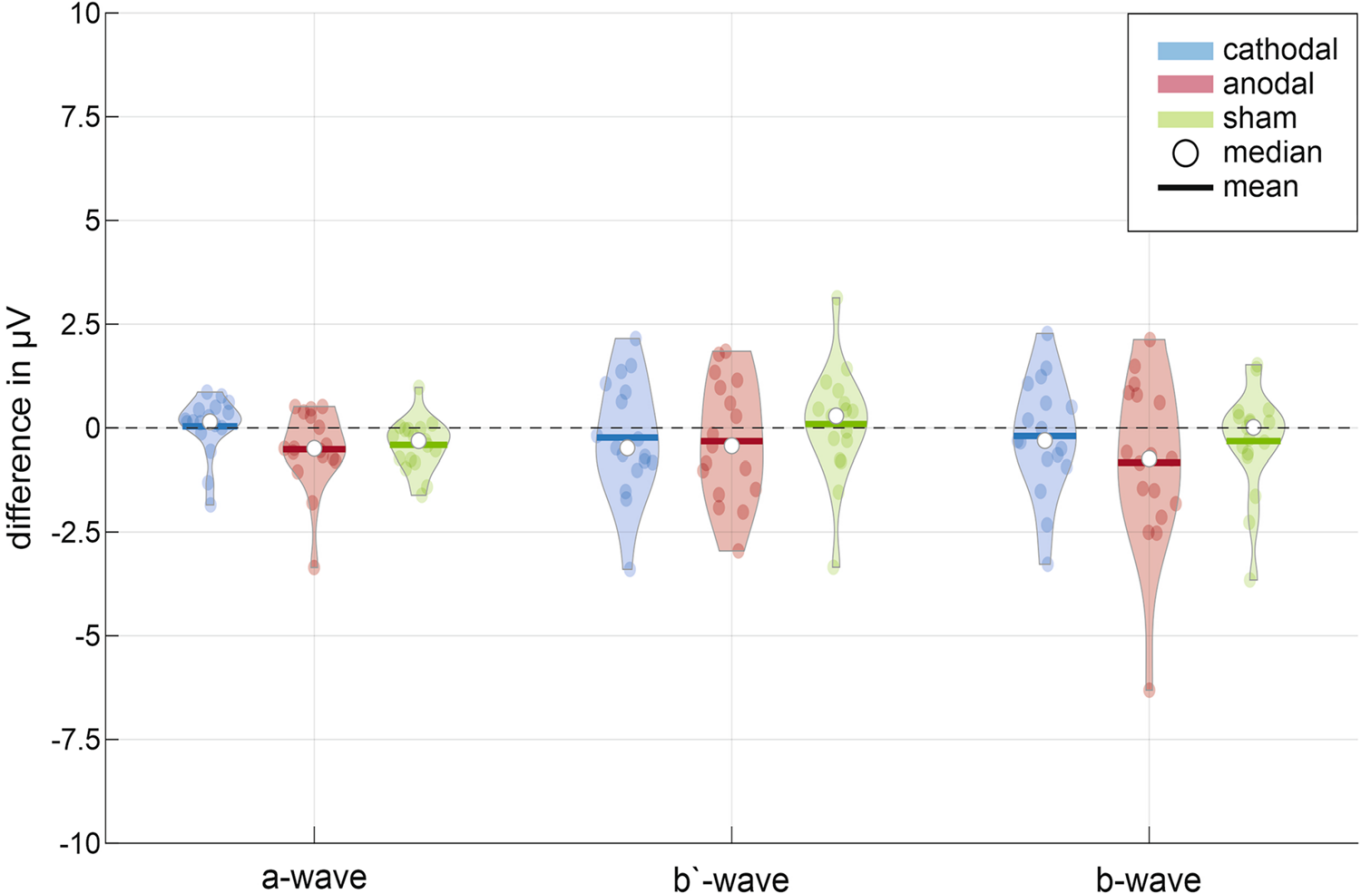
Data distribution full field ERG waves. Violin plots show the data distribution of the difference between the ERG 1 and ERG 2 measurement (ERG 1 before, ERG 2 during ocular current stimulation) for the characteristic a-wave (first minimum), b’-wave (maximum measured from zero line) and b-wave (peak-to-peak measurement) and current stimulation groups.

The data distribution of the a-, b’- and b-wave for the cathodal and anodal oCS showed no sign of significant difference to the results of the sham-stimulation (Fig. 3). Therefore, a further statistical evaluation for these characteristic waves was waived.

## Discussion

In the present study, the effect of oCS on the ffERG characteristic PhNR wave was tested to investigate the influence on the summed activity of retinal ganglion cells. Therefore, a new measurement and stimulation setup was designed, with which the PhNR wave could be measured before (ERG 1) and during (ERG 2) direct current stimulation of the eye for three current applications (i.e., cathodal polarity, anodal polarity, or sham stimulation). We found a polarity-dependent oCS effect on the PhNR amplitude (cathodal increasing, anodal decreasing, sham stimulation no effect).

The PhNR wave is assigned to the activity of the ganglion cells^34,40^ and is comparable to the characteristic waves of the PERG. In a PERG study^30^, the authors found a polarity-independent oCS effect on the P50 amplitude, which was probably regulated by ganglion cells^31^. In the present PhNR study, a significant oCS effect on the cathodal PhNR amplitude and between the cathodal group and the two other stimulation groups was found. This confirmed that oCS influences retinal ganglion cells.

From VEP studies in combination with simultaneous tDCS of the visual cortex, it is known that polarity-dependent influences on the characteristic VEP amplitudes occur^3–5^. The VEP reflects an electrophysiological sum response of activated neurons in the visual cortex. In the present PhNR study, a significant difference in PhNR amplitude changes was observed between the cathodal and anodal oCS groups (cathodal increasing, anodal decreasing). Furthermore, the sham stimulation group showed no oCS effect. Consequently, the present study result is consistent with the outcome from VEP studies^3–5^. This confirmed the possibility of a polarity-dependent influence from oCS on ganglion cell activity. Due to the fact that the measured ERG was a full field ERG we didn’t expected the characteristic a, b’, and b-wave to show other results than published in Blum et al.^32^. This was confirmed by the present study, thus reinforcing the hypothesis that ganglion cells can be influenced by the performed oCS.

In the PERG study, ganglion cell activity decreased independently of oCS polarity^30^, while in the present study, the PhNR amplitude increased or decreased depending on the polarity of the oCS. This difference in oCS polarity dependences can be explained by the different origins of the characteristic ERG waves in the ganglion cells between PERG and PhNR. The PERG is a calculation of the local ON and OFF cell activity differences of the ganglion cells. The P50 wave represents the input activity in ganglion cells influenced by preganglion cells, while the N95 wave represents ganglion cell spiking activity^40^. In contrast, the PhNR represents the spiking activity of ganglion cells as a sum response^34,38^. The fact that the PhNR amplitude can be influenced in a polarity-dependent manner while the PERG is affected independent of the current polarity supports the hypothesis that the oCS effect on the P50 amplitude is caused by different local ON and OFF cell activities. Theoretically, both the N95 amplitude of the PERG and the PhNR amplitude of an ffERG represent the spiking activity of retinal ganglion cells, whereupon it could be expected that both amplitudes should be equally influenced with respect to oCS polarity. However, the authors found no oCS effect on the N95 amplitude of the PERG^30^. Since the PERG is the differential signal between the ON and OFF responses, cancellation effects cannot be excluded. Based on the present study, no reliable conclusion can be made regarding the effect of oCS on the different ganglion cell types. To further investigate this topic, possible approaches to selectively stimulate ganglion cell types will be based on the administration of drugs^41^ or the examination of patients who have a disorder in a specific ganglion cell type. Furthermore, special variations in the visual stimulation parameters, e.g., ON–OFF ERGs^42^, or adaptations are conceivable.

The newly designed study setup enabled the recording and evaluation of the PhNR wave for all subjects and measurement conditions, including simultaneous oCS. Furthermore, due to the use of skin electrodes and transorbital current application, the study was volunteer friendly. Typical side effects such as corneal epithelial damage or worsening dry eye symptoms could be avoided. It must be noted that the setup is a compromise between the requirements for visual stimulation, PhNR measurement and oCS. The cutout within the oCS electrode surrounding the eye contradicted the requirement for a homogeneous current injection. This cutout was necessary to position the noninvasive ERG skin electrode at the lower eyelid without a galvanic or wire connection to the oCS electrode. Smaller electrode surfaces for the oCS would have contradicted the requirement of a small current density at the sensitive skin surrounding the eye. Therefore, the ERG reference electrode could not be positioned at the ipsilateral canthus, as suggested by the ISCEV standard. For future work, research on electrode techniques and attachment possibilities is therefore of great interest.

The study is limited by the current stimulation parameters, the stimulation electrode positions and the number of volunteers. The study was designed with an a priori power analysis based on a preliminary study^33^ (α = 0.05, β = 0.2, d = 0.7, nonparametric test). The power of the cathodal stimulation group was 0.92 based on an effect size of d_cathodal_ = 0.77, the inclusion of 17 volunteers and the possibility of using a parametric statistical test. Consequently, a higher number of volunteers would be necessary to be able to detect or reject smaller effect sizes, such as d_anodal_ = 0.55, as significant. Furthermore, the current stimulation effects depend on the neuronal morphology relative to the generated electrical field^43^. Therefore, the positions of the current stimulation electrodes are crucial for the effects of the current stimulation. It is conceivable that the positioning of the current stimulation electrodes at the eye and the ipsilateral tempus would be better suited for stimulating ganglion cells than other retinal cells. Repositioning the stimulation electrode from the ipsilateral temple, for example, to the Oz position would generate a more homogeneous current flow through the entire eye^44^ and could therefore produce other effects. The relation between the stimulation parameters and the occurring electrophysiological and neurological effects is nonlinear^45^. Therefore, the use of other current stimulation parameters besides the 800 µA over 5 min in this study could lead to other results. Different current electrode positions and parameter variation studies should be modelled and analyzed in the future^46^.

In conclusion, we designed a working measurement and stimulating setup with which we found a significantly increasing PhNR amplitude during cathodal oCS. In addition, the PhNR amplitude changes differed significantly between the cathodal and anodal groups as well as between the cathodal and sham stimulation groups. This finding indicates that retinal ganglion cells can be influenced by oCS in a polarity-dependent manner. Further investigations should address the separation of individual ganglion cell types as well as the variation in stimulation parameters.

## Material and methods

### Participants

Seventeen healthy volunteers (mean age: 25.2 ± 3.1 years, 9 females) participated in the study, which was permitted by the Ethics Commission at the medical faculty of Friedrich-Schiller-University Jena, Germany. Participation was voluntary. All volunteers gave their written informed consent according to the Declaration of Helsinki to participate in this study. Discontinuation was possible at any time. Consent for data processing could be revoked at any time. The exclusion criteria were as follows: neurological, eye, skin, or heart diseases; metal implants in the head area; allergies or hypersensitivities of the skin; pregnancy; and ametropia >|2| diopters. The volunteers were invited to three independent sessions (randomized order) with different current applications (i.e., cathodal polarity, anodal polarity, or sham stimulation). All measurements were conducted by the same individual.

### Measuring and stimulation setup

A new measurement and stimulation setup was developed to enable simultaneous current stimulation and ffERG measurement adjusted to the special requirements for measurement of the PhNR. One general requirement was the avoidance of invasive procedures for all parts of the setup. Furthermore, the following technical aspects had to be observed.

A field stimulator with different spectral stimulations and backlights as well as different luminance opportunities was required for visual stimulation. The biosignal amplifier had to be galvanically isolated from all other components of the measurement and stimulation setup. The amplifier needed to have a high dynamic range as well as a high-resolution analog-to-digital converter (24-bit). This was necessary to compensate the resulting offset originating from the direct current stimulation and at the same time provides good amplification and resolution to measure small effect sizes in the ERG. For simultaneously measuring the ERG with current stimulation, Ag/AgCl electrodes should be used because of their long-term stability, low-frequency noise and stability against polarization effects^47^. The current stimulator had to have a low noise behavior, must be operable in battery mode, and must be able to measure the impedance between the stimulation electrodes over the entire duration. The impedance between the current stimulation electrodes must be kept as low as possible because additional noise occurs depending on the size of the impedance, which will interact with the ERG. Furthermore, an electrode attachment had to be chosen such that it allowed a safe, homogeneous, and evenly distributed current injection into the eye as well as a small current density to protect the sensitive skin and nerves around the eye. In addition, it must be possible to attach the ERG measuring electrodes at and around the eye with no galvanic connection or wire interference to the current stimulation electrodes.

There were three current applications: cathodal polarity, anodal polarity, and sham stimulation. The applied current polarity (cathodal or anodal) refers to the polarity at the stimulation electrode surrounding the eye. During the sham stimulation, no current flow (current source not activated) was generated at the electrodes, but the volunteers were informed that current stimulation was performed. Due to randomization and unawareness of the subjects that one of the three sessions will be a sham stimulation, the participants could not distinguish which current form was applied based on individual reports.

### Experimental timeline

**Figure 4:**
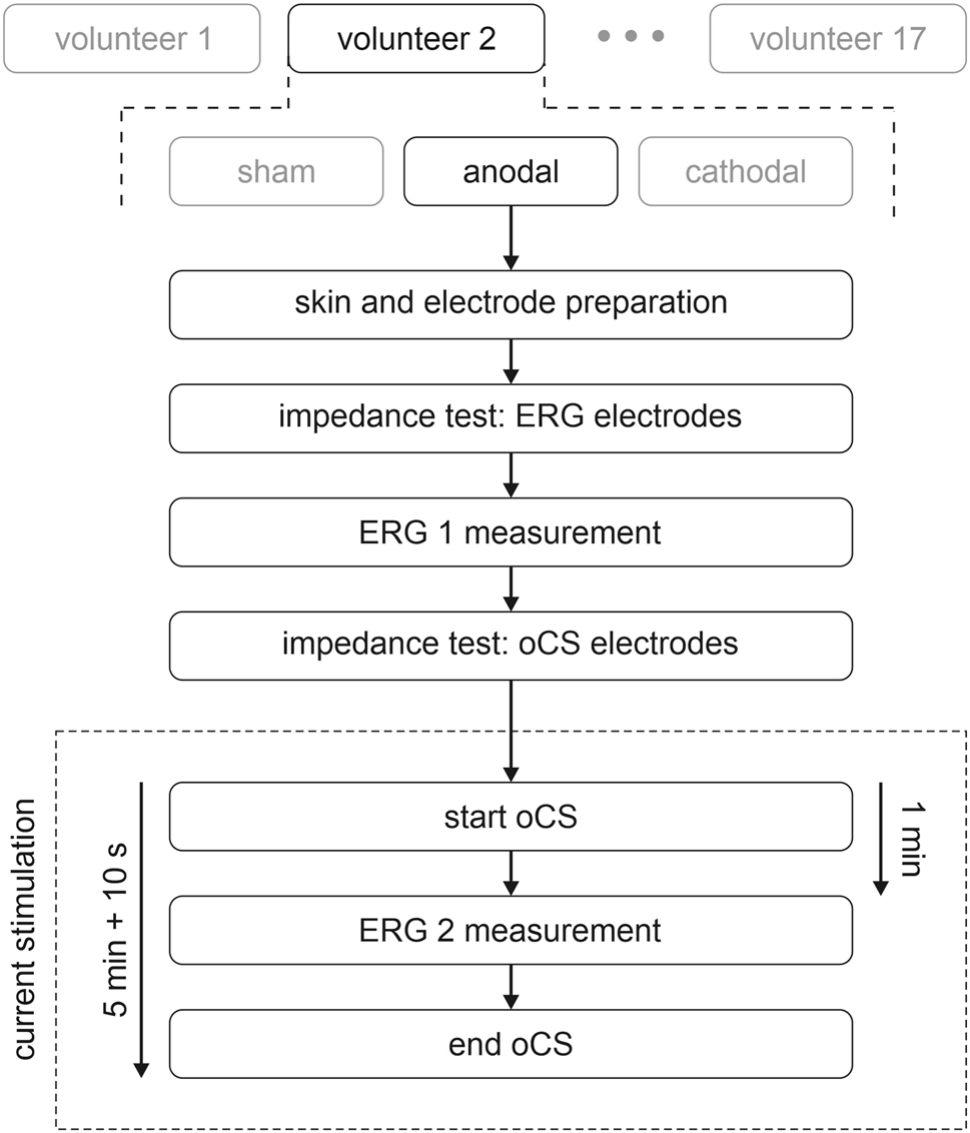
Measurement procedure. Every volunteer went through three sessions (randomized order, different days) which differed in the type of the ocular current stimulation (oCS): cathodal polarity, anodal polarity, or sham stimulation. In each session two ERG measurements were performed, one before (ERG 1) and one during (ERG 2) the oCS. The current stimulation lasted 5 min plus 5 s Fade-In and 5 s Fade-Out time, respectively.

In total, every volunteer went through three sessions conducted on different days with at least one day between sessions. Each session comprised two separate monocularly (9 right eyes, 8 left eyes) ERG recordings: one before (ERG 1) and one during (ERG 2) the current stimulation. The sessions differed only in the type of current application (cathodal polarity, anodal polarity, or sham stimulation). First, the skin was cleaned with alcohol, and the positions for the ERG electrodes were prepared with contact gel (NuPrep, Weaver and Company, Aurora, Colorado, United States) to ensure good signal quality. Furthermore, the volunteer’s hair was slightly moistened with saline solution before applying the current stimulation electrode to achieve a low electrode impedance. All electrodes were stabilized with tape, and the current stimulation electrodes were additionally kept in place by a fixation strap. After preparing and attaching all electrodes, an impedance test was performed for the ERG electrodes. Here, impedances ≤ 15 kΩ and a difference ≤ 5 kΩ between the ERG electrode impedances were admitted. When the necessary impedance values were reached, the volunteers adapted for 15 s to the blue stimulation background, and then the ERG 1 measurement was performed. In total, 250 single flashes were presented per measurement. Subsequently, an impedance test (sinus alternating current, 200 µA, 20 Hz) was carried out for the current stimulation electrodes. To start the current stimulation, the impedance had to be ≤ 8 kΩ. The adaptation time of 15 s to the blue background started 45 s after the start of oCS so that 1 min after the start of oCS, the ERG 2 measurement was performed. Figure 4 illustrates the study procedure. In order to create absolutely identical light conditions, during the measurements the examination room was darkened to allow adaptation to the blue background without bias due to preconditions. Between the measurements, the room was lightened again.

### Signal processing

Signal processing was performed with MATLAB (version 2020b, The Mathworks, Inc., Natick, United States). First, the raw ERG signal was filtered forward and backward to avoid phase shifting with an infinite impulse response (IIR) high pass (Butterworth, filter order: 3, half power frequency: 0.75 Hz) and low pass (Butterworth, filter order: 10, half power frequency: 70 Hz) filter. Sweeps that contained amplitudes higher than ± 100 µV were evaluated as artifact afflicted and excluded from further signal processing. For each of the remaining sweeps, the Pearson correlation was calculated with the mean over all artifact-free sweeps. The 200 sweeps with the highest correlation coefficients were averaged. The averaged signal was centered onto the zero point (time and amplitude zero). The PhNR wave was defined as the minimum wave between 60 ms and 80 ms after stimulus onset and was measured from the zero line. Furthermore, the a-wave (first minimum, measured from zero line), b-wave (maximum, measured from the a-wave peak), and the b’-wave (b-wave peak measured from zero line) were determined.

### Statistical analysis

Statistical analysis was performed using IBM SPSS Statistics (version 25, IBM Corp., Armonk, United States). The study was designed with an a priori power analysis for a nonparametric test with a significance level of α = 0.05, a power of (1-β) = 0.8, and an effect size according to a Cohen’s d^39^ of d = 0.7, estimated on the basis of a preliminary study^33^.

The primary aim of the study was to identify direct current stimulation effects on the PhNR amplitude by analyzing the difference between the ERG 1 and ERG 2 measurements. The normal distribution hypothesis was not rejected by the Shapiro–Wilk test and Q-Q plot (see Supplementary Table S1, Fig. S1). Therefore, the t-test for related samples, including a confidence interval analysis, was performed for every current application group. Based on the multiple comparison problem, the Bonferroni correction resulted in an adjusted significance value of p* ≤ 0.0167. The resulting effect size of the present study outcome was determined using Cohen’s d^39^.

The secondary study aim was to analyze the difference between the three current application groups for the change from the ERG 1 to the ERG 2 measurement. Therefore, repeated-measures ANOVA was performed. As a post hoc test, the t-test for related samples was used with Bonferroni correction.

Grand mean curves and violin plots were plotted to allow a graphical analysis and evaluation of the data distribution for every stimulation group, ERG measurement, and characteristic ERG wave.

## Supplementary Information

Supplementary Information.

## References

[1] Lefaucheur J-P (2017). Evidence-based guidelines on the therapeutic use of transcranial direct current stimulation (tDCS). Clin. Neurophysiol..

[2] Yokoi Y, Sumiyoshi T (2015). Application of transcranial direct current stimulation to psychiatric disorders: Trends and perspectives. Neuropsychiatr. Electrophysiol..

[3] Antal A, Kincses TZ, Nitsche MA, Bartfai O, Paulus W (2004). Excitability changes induced in the human primary visual cortex by transcranial direct current stimulation: Direct electrophysiological evidence. Investig. Opthalmology Vis. Sci..

[4] Accornero N, Li Voti P, La Riccia M, Gregori B (2007). Visual evoked potentials modulation during direct current cortical polarization. Exp. Brain Res..

[5] Ding Z (2016). The effect of transcranial direct current stimulation on contrast sensitivity and visual evoked potential amplitude in adults with amblyopia. Sci. Rep..

[6] Frase L (2021). Transcranial direct current stimulation induces long-term potentiation-like plasticity in the human visual cortex. Transl. Psychiatry.

[7] Wunder S, Hunold A, Fiedler P, Schellhorn K, Haueisen J (2018). Novel bifunctional cap for simultaneous electroencephalography and transcranial electrical stimulation. Sci. Rep..

[8] Chow AY (2004). The artificial silicon retina microchip for the treatment of vision loss from retinitis pigmentosa. Arch. Ophthalmol..

[9] Morimoto T, Miyoshi T, Matsuda S, Tano Y (2005). Transcorneal electrical stimulation rescues axotomized retinal ganglion cells by activating endogenous retinal IGF-1 s ystem. Investig. Ophthalmol. Vis. Scince.

[10] Morimoto T (2007). Transcorneal electrical stimulation promotes the survival of photoreceptors and preserves retinal function in Royal College of Surgeons rats. Investig. Ophthalmol. Vis. Sci..

[11] Morimoto T (2012). Transcorneal electrical stimulation promotes survival of photoreceptors and improves retinal function in rhodopsin P347L transgenic rabbits. Investig. Ophthalmol. Vis. Sci..

[12] Schatz A (2012). Transcorneal electrical stimulation shows neuroprotective effects in retinas of light-exposed rats. Investig. Ophthalmol. Vis. Sci..

[13] Tagami Y (2009). Axonal regeneration induced by repetitive electrical stimulation of crushed optic nerve in adult rats. Jpn. J. Ophthalmol..

[14] Sehic A (2016). Electrical stimulation as a means for improving vision. Am. J. Pathol..

[15] Gil-Carrasco F (2018). Transpalpebral electrical stimulation as a novel therapeutic approach to decrease intraocular pressure for open-angle glaucoma: A pilot study. J. Ophthalmol..

[16] Ota Y (2018). The efficacy of transcorneal electrical stimulation for the treatment of primary open-angle glaucoma: A pilot study. Keio J. Med..

[17] Jolly JK (2020). Transcorneal electrical stimulation for the treatment of retinitis pigmentosa: A multicenter safety study of the OkuStim® System (TESOLA-Study). Ophthalmic Res..

[18] Schatz A (2011). Transcorneal electrical stimulation for patients with retinitis pigmentosa: A prospective, randomized, sham-controlled exploratory study. Investig. Ophthalmol. Vis. Scince.

[19] Schatz A (2017). Transcorneal electrical stimulation for patients with controlled follow-up study over 1 year. Investig. Ophthalmol. Vis. Scince.

[20] Wagner SK (2017). Transcorneal electrical stimulation for the treatment of retinitis pigmentosa: Results from the TESOLAUK trial. BMJ Open Ophthalmol..

[21] Röck T (2013). Transkorneale Elektrostimulation bei Patienten mit Morbus Stargardt. Der Ophthalmol..

[22] Anastassiou G, Schneegans A-L, Selbach M, Kremmer S (2013). Transpalpebral electrotherapy for dry age-related macular degeneration (AMD): An exploratory trial. Restor. Neurol. Neurosci..

[23] Chaikin L, Kashiwa K, Bennet M, Papastergiou G, Gregory W (2015). Microcurrent stimulation in the treatment of dry and wet macular degeneration. Clin. Ophthalmol..

[24] Shinoda K (2008). Transcutaneous electrical retinal stimulation therapy for age-related macular degeneration. Open Ophthalmol. J..

[25] Inomata K (2007). Transcorneal electrical stimulation of retina to treat longstanding retinal artery occlusion. Graefe’s Arch. Clin. Exp. Ophthalmol..

[26] Naycheva L (2013). Transcorneal electrical stimulation in patients with retinal artery occlusion: A prospective, randomized sham-controlled pilot study. Ophthalmol. Ther..

[27] Fedorov A (2011). Restoration of vision after optic nerve lesions with noninvasive transorbital alternating current stimulation: A clinical observational study. Brain Stimul..

[28] Gall C (2011). Noninvasive transorbital alternating current stimulation improves subjective visual functioning and vision-related quality of life in optic neuropathy. Brain Stimul..

[29] Sabel BA, Fedorov AB, Naue N, Borrmann A (2011). Non-invasive alternating current stimulation improves vision in optic neuropathy. Restor. Neurol. Neurosci..

[30] Blum M-C, Hunold A, Solf B, Klee S (2020). The effects of an ocular direct electrical stimulation on pattern-reversal electroretinogram. Front. Neurosci..

[31] Blum M-C, Solf B, Hunold A, Klee S (2021). Effects of ocular direct current stimulation on full field electroretinogram. Front. Neurosci..

[32] Frishman, L. J. Origin of the electroretinogram. In *Principles and Practice of Clinical Electrophysiology of Vision* 139–184 (2006).

[33] Blum, M.-C., Solf, B. and Klee, S. Photopic negative response during and after ocular direct current stimulation. Acta Ophthalmol. 99, (2021).

[34] Frishman, L. J. & Wang, M. H. Electroretinogram of Human, Monkey and Mouse. In *Adler’s physiology of the eye* 480–501 (2011).

[35] Frishman LJ (2018). ISCEV extended protocol for the photopic negative response (PhNR) of the full-field electroretinogram. Doc. Ophthalmol..

[36] Machida S (2012). Clinical applications of the photopic negative response to optic nerve and retinal diseases. J. Ophthalmol..

[37] Rangaswamy NV (2007). Effects of spectral characteristics of ganzfeld stimuli on the photopic negative response (PhNR) of the ERG. Investig. Ophthalmol. Vis. Sci..

[38] Viswanathan S, Frishman LJ, Robson JG, Harwerth RS, Smith EL (1999). The photopic negative response of the macaque electroretinogram: Reduction by experimental glaucoma. Investig. Ophthalmol. Vis. Sci..

[39] Cohen J (1988). Statistical Power Analysis for the behavioral Sciences.

[40] Bach, M. & Hoffmann, M. B. The origin of the pattern electroretinogram. In *Principles and Practice of Clinical Electrophysiology of Vision* 185–196 (2006).

[41] Luo X, Frishman LJ (2011). Retinal pathway origins of the pattern electroretinogram (PERG). Investig. Ophthalmol. Vis. Sci..

[42] Sustar M (2018). ISCEV extended protocol for the photopic On–Off ERG. Doc. Ophthalmol..

[43] Bikson, M., Paulus, W., Esmaeilpour, Z., Kronberg, G. & Nitsche, M. A. Mechanisms of acute and after effects of transcranial direct current stimulation. In *Practical Guide to Transcranial Direct Current Stimulation* 81–113 (2019).

[44] Hunold A, Freitag S, Schellhorn K, Haueisen J (2015). Simulation of the current density distribution for transcranial electric current stimulation around the eye. Brain Stimul..

[45] Jamil A (2017). Systematic evaluation of the impact of stimulation intensity on neuroplastic after-effects induced by transcranial direct current stimulation. J. Physiol..

[46] Sabel BA (2020). Vision modulation, plasticity and restoration using non-invasive brain stimulation: An IFCN-sponsored review. Clin. Neurophysiol..

[47] Woods AJ (2015). A technical guide to tDCS and related non-invasive brain stimulation tools. Clin. Neurophysiol..

