# Supplementary material for: Ocular direct current stimulation affects retinal ganglion cells
Source: Sci Rep. 2021 Sep 2;11:17573. doi: 10.1038/s41598-021-96401-9 (PMC8413326; doi:10.1038/s41598-021-96401-9)
Supplement: Supplementary file 1 — Supplementary Information. [file 41598_2021_96401_MOESM1_ESM.docx]

**Ocular direct current stimulation affects retinal ganglion cells**

Maren-Christina Blum^1*^, Alexander Hunold^1^, Benjamin Solf ^1^, Sascha Klee^1,2^

^1^Institute for Biomedical Engineering and Informatics, Technische Universität Ilmenau, Ilmenau, Germany

^2^Department of General Health Studies, Division Biostatistics and Data Science, Karl Landsteiner University of Health Sciences, Krems, Austria

***** maren.blum@tu-ilmenau.de

**Supplementary Material**

**Table S1**: Results of the Shapiro-Wilk test to test whether the amplitude values of the ERG 1 and ERG 2 measurements and the difference between them are normal distributed. The significance level was set to α = 0.05.

| stimulation group | measurement | p-value |
| --- | --- | --- |
| anodal | ERG 1 | 0.809 |
|  | ERG 2 | 0.841 |
|  | difference | 0.852 |
| cathodal | ERG 1 | 0.202 |
|  | ERG 2 | 0.938 |
|  | difference | 0.810 |
| sham | ERG 1 | 0.597 |
|  | ERG 2 | 0.088 |
|  | difference | 0.243 |

**Figure S1**: QQ-diagrams of the photopic negative response (PhNR) amplitudes. QQ-diagrams of the measured PhNR amplitudes of the ERG 1 and ERG 2 measurement and the difference between them for the three different current application groups (i.e., cathodal polarity, anodal polarity, and sham-stimulation) to test whether the measurement values are normal distributed.
